# Microfluidic device engineered to study the trafficking of multiple myeloma cancer cells through the sinusoidal niche of bone marrow

**DOI:** 10.1038/s41598-022-05520-4

**Published:** 2022-01-27

**Authors:** Chao Sui, Jenny Zilberberg, Woo Lee

**Affiliations:** 1 Department of Chemical Engineering and Materials Science, Stevens Institute of Technology, 1 Castle Point on Hudson, Hoboken, NJ 07030 USA; 2 Hackensack Meridian Health, Center for Discovery and Innovation, Nutley, NJ 07110 USA; 3 Department of Chemistry and Chemical Biology, Stevens Institute of Technology, 1 Castle Point On Hudson, Hoboken, NJ 07030 USA

**Keywords:** Lab-on-a-chip, Bone cancer

## Abstract

Multiple myeloma (MM) is an incurable B cell malignancy characterized by the accumulation of monoclonal abnormal plasma cells in the bone marrow (BM). It has been a significant challenge to study the spatiotemporal interactions of MM cancer cells with the embedded microenvironments of BM. Here we report a microfluidic device which was designed to mimic several physiological features of the BM niche: (1) sinusoidal circulation, (2) sinusoidal endothelium, and (3) stroma. The endothelial and stromal compartments were constructed and used to demonstrate the device’s utility by spatiotemporally characterizing the CXCL12-mediated egression of MM cells from the BM stroma and its effects on the barrier function of endothelial cells (ECs). We found that the egression of MM cells resulted in less organized and loosely connected ECs, the widening of EC junction pores, and increased permeability through ECs, but without significantly affecting the number density of viable ECs. The results suggest that the device can be used to study the physical and secreted factors determining the trafficking of cancer cells through BM. The sinusoidal flow feature of the device provides an integral element for further creating systemic models of cancers that reside or metastasize to the BM niche.

## Introduction

Significant progress has been made with developing microfabricated cell culture devices to recapitulate in vitro the physiological functions of organs (“organs on a chip”) and model the pathophysiological developments of human diseases^1^. This research field has been largely driven over the past decade to address various shortcomings of animal models in capturing human diseases, particularly for predicting the safety and efficacy of drugs and biomaterials^2,3^. Organs-on-a-chip are typically developed by spatiotemporal control of organ-specific biological, chemical, and physical factors of native tissue microenvironments, therefore mimicking physiological functions of tissue cells. Our long-term interest^4–8^ has been to recapitulate in vitro the physiological features of the human bone marrow (BM) microenvironments and investigate how these microenvironments contribute to the dormancy, survival, drug resistance, and opportunistic progression of human multiple myeloma (MM) cells.

**Figure 1:**
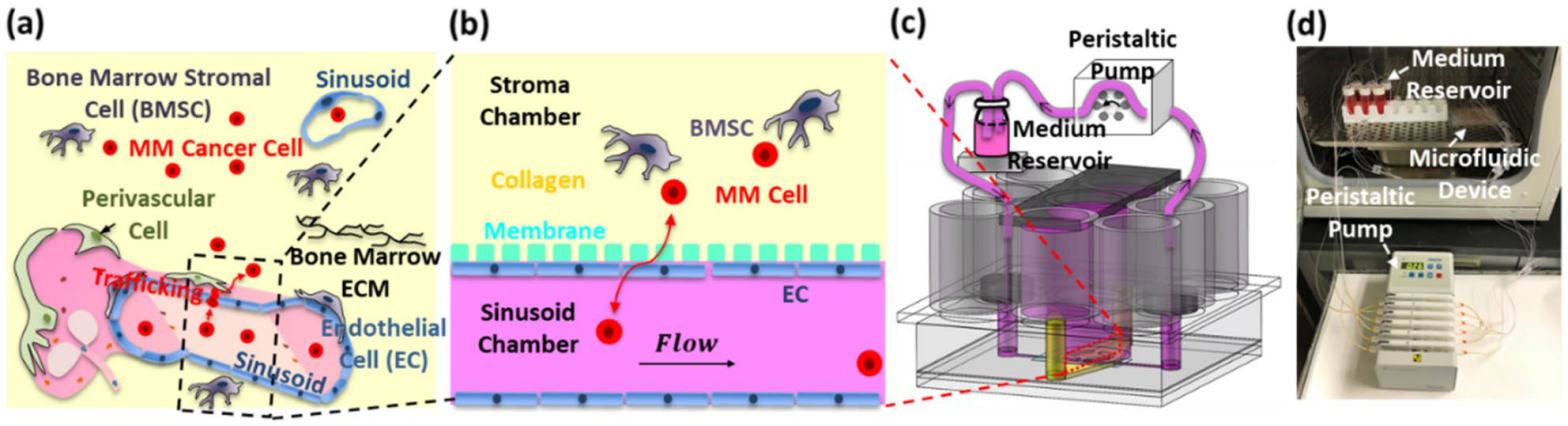
Microfluidic culture device designed to mimic the trafficking of cancer cells through the sinusoidal niche of bone marrow (BM). (**a**) Schematic illustration of major physiological features of the sinusoidal niche. (**b**) Schematic illustration of the recapitulated sinusoidal niche in the device. (**c**) Device design based on a 96-well plate configuration. (**d**) Actual device operated inside an incubator using an external peristaltic pump.

MM is a B-cell malignancy characterized by: (1) the accumulation of monoclonal abnormal plasma cells in BM and (2) the progression of osteolytic lesions^9^. Despite much progress in the treatment of cancer, MM remains an incurable disease^10,11^. Devastatingly, most MM patients experience relapses due to the development of drug-resistant cancer cells and have a typical post diagnosis survival of only 5 to 7 years^12^. Similarly, breast and prostate cancers are incurable once cancer cells are established within the BM^13,14^. During active progression, MM cells can be found in blood circulation and disseminated from one BM site to another^15^. As illustrated in Fig. 1a, the homing of MM cells from peripheral blood to BM is known to be driven by the chemoattract CXCL12 (10 kDa) expressed by BM cells, including mesenchymal stem cells (MSCs), BM stromal cells (BMSCs), and osteoblasts (OSBs)^16^. MM cells express the chemokine receptor CXCR4, and are therefore attracted to CXCL12^+^ cells in the BM. Conversely, the egress of MM cells from the BM is dependent on overcoming this CXCL12/CXCR4 retention signal (e.g., decreased CXCR4 expression of MM cells), and is associated with increased dissemination and poor prognosis^17^. Clinically, treatment with AMD3100 (a CXCR4 inhibitor) leads to mobilization of MM cells from the BM to the peripheral blood^18^.

The BM is highly vascularized by sinusoid microvessels to support the trafficking of blood and immune cells (e.g., billions of lymphocytes trafficking through BM in the human body every day)^19^. The microvascular system of the BM is distinguished from those in other tissues by its irregular network of interconnecting arteriolar and sinusoidal microvessels^20^. The lumen of sinusoidal microvessels is composed of a porous and leaky layer of endothelial cells (ECs), allowing the trafficking of leukocytes and hematopoietic stem and progenitor cells^21,22^. This leakiness allows the trafficking of immune cells. Cell trafficking into the circulation was found to occur where sinusoidal microvessels are solely composed of ECs, without the perivascular fibrous layer of adventitial cells^23^. The transmigration of BM cells through sinusoidal endothelium appears to occur mainly through small pores of about 1.2 to 2 μm located at sinusoidal EC junctions^23,24^. Furthermore, intravital microscopy studies^20,24^ have shown that the average velocity of blood through sinusoidal microvessels (~ 20 µm in diameter) is on the order of ~ 0.2 mm/s with the corresponding shear stress (*τ*_*w*_) of ~ 0.1 Pa exerted on ECs.

The goal of this study was to develop a new microfluidic culture device that can be used to study the trafficking of MM cells through the sinusoidal niche of the BM. As illustrated in Fig. 1b, our design approach was to mimic: (1) the sinusoidal blood circulation in the BM, represented by flow of the culture medium in the sinusoid chamber with a physiological *τ*_*w*_ of ~ 0.1 Pa; (2) the sinusoidal endothelium of BM, represented by a monolayer of ECs; and (3) BM stroma, represented by fibroblastic BMSCs dispersed in collagen in the stroma chamber. The first objective was to design and fabricate the device by following our previously established practice^7,25^ of using industry standard 96-well plate format (Fig. 1c). The second objective was to reconstruct the endothelium and BM stroma and assess the endothelium’s barrier function by measuring permeability. The third objective was to demonstrate the device’s utility by spatiotemporally characterizing the CXCL12-mediated egression of MM cells from the BM stroma and its effects on the endothelium’s morphology and barrier function. Importantly, the effects of MM cell egression on the sinusoidal endothelium barrier functions have not been reported to our best knowledge.

## Results

### Device design and operation

**Figure 2:**
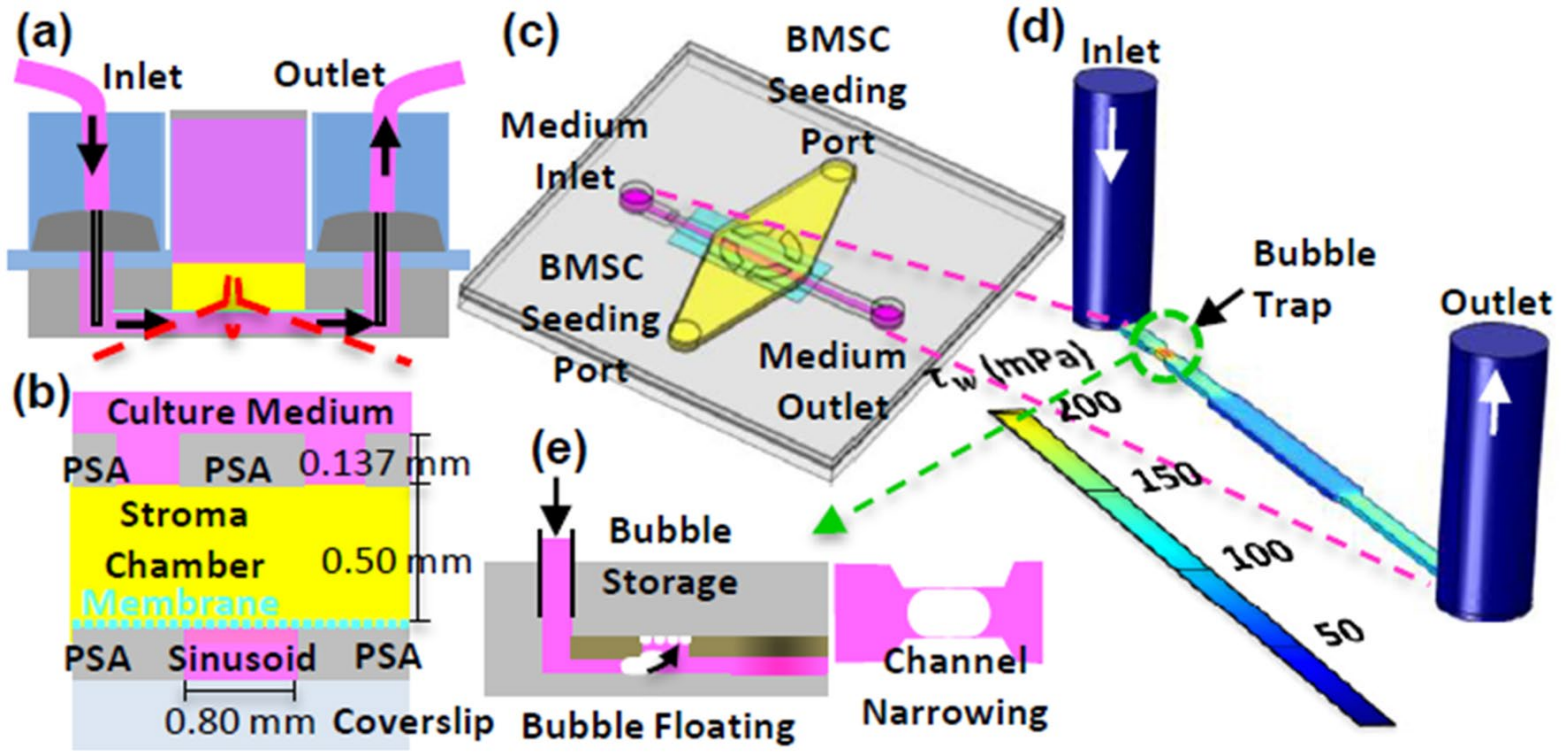
Design features of the sinusoid and stroma chambers. (**a**) and (**b**) Cross-sectional views of the device. (**c**) Top view of the device. (**d**) Simulated shear stress contours along the axial direction of the sinusoid chamber. (**e**) Bubble trap via chamber narrowing and storage along the sinusoid chamber.

The sinusoidal blood flow was mimicked using a peristaltic pump and an external medium reservoir, as we previously reported^5^, to recirculate the medium (Fig. 1c,d) and generate *τ*_*w*_ through the rectangular sinusoid chamber (0.8 mm width and 0.137 mm depth) on the order of 0.1 Pa. COMSOL Multiphysics 5.3 was used to compute the velocity profiles and shear stress distribution in the sinusoid chamber (Fig. 2d). The medium flow was simulated as a steady state and laminar flow using the following parameters: volumetric flow rate (8.98 µL/min), density (10^3^ kg/m^3^), and dynamic viscosity (0.9 mPa s). The simulation (Fig. 2d) shows that the shear stress distribution can be controlled to be relatively uniform along the sinusoid chamber with a wall shear stress of 0.094 Pa. A bubble trap was placed before the sinusoid chamber (Fig. 2e) to mitigate the propensity for bubble introduction due to the high-velocity flow of the culture medium. It was designed to increase fluidic resistance by narrowing the chamber width while providing an additional volume above the chamber for bubbles to be stored^26^.

The sinusoidal endothelium was mimicked by placing a transparent polyester (PETE) membrane between the sinusoid and stroma chambers (Figs. 1b, 2a–c) and establishing a confluent layer of ECs on the bottom surface of the membrane. In this design, the membrane provided: (1) mechanical support for the stroma chamber, (2) mechanical protection of the collagen scaffold in the stroma chamber from the high-velocity flow in the sinusoid chamber, (3) a surface for EC adhesion and growth, and (4) open pores of 10 μm to allow the migration of MM cells through the reconstructed endothelium. The culture medium ports (Fig. 2a,c) were used to introduce ECs into the sinusoid chamber.

**Figure 3:**
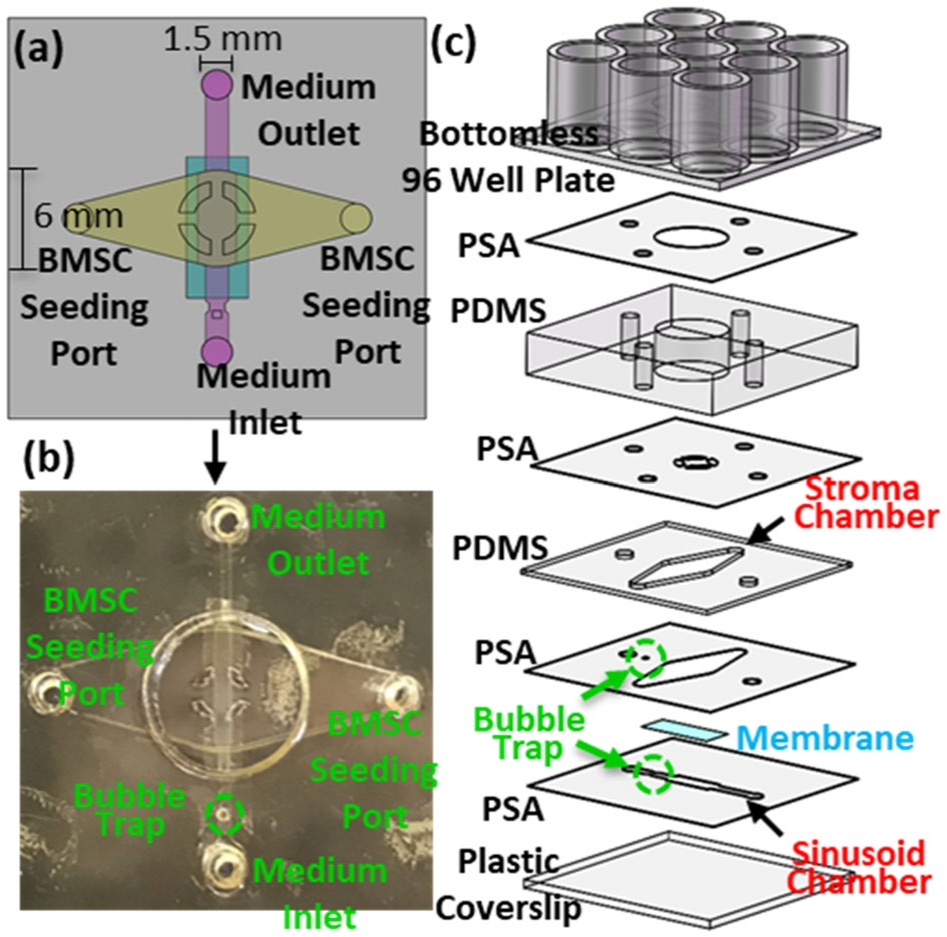
Device fabrication. (**a**) Schematic top view of one pair of the sinusoid and stroma chambers. (**b**) Picture of the actual device fabricated prior to assembly onto a bottomless 96-well plate. (**c**) Schematic illustration of the layer-by-layer assembly used to produce the microfluidic device.

The stroma chamber (i.e., the yellow compartment in Figs. 1b and 2a–c) was designed to culture BMSCs dispersed in collagen (volume = 60 mL). In this design, collagen was used to represent the viscoelastic extracellular matrix of the BM. Its elongated shape provided a large surface area and surface tension to minimize the remodeling and contraction of collagen upon culture. The medium reservoir placed above the stroma chamber (Figs. 2a, 3b) was designed to: (1) provide periodic replenishment of culture medium by pipetting during long-term culture, (2) prevent collagen overflow above the stroma chamber during the BMSC seeding step, and (3) introduce stains and dyes for convenient endpoint characterization. Nine wells were used to construct and integrate the sinusoid and stroma chambers, thus enabling six culture experiments to be conducted within each microfluidic device.

The microfluidic device (Figs. 3 and S1) was fabricated using previously reported techniques^5,7,25^. Details of the device fabrication procedures are provided in Methods and Supplementary Information 1. As shown in Figs. 1c and 3c, 9 wells of the well-plate were used to produce one pair of sinusoid and stroma chambers and therefore 6 pairs for each device. Therefore, each device could be used to perform 6 culture experiments. As shown in Fig. 1d, 6 sinusoid chambers in each device were connected to 6 external culture medium reservoirs and 6 out of 12 flow channels available in the peristaltic pump (IP ISM942, ISMATEC) using external tubing. Fluidic connections with the devices and external tubes were made using two polydimethylsiloxane (PDMS) pieces inserted into the inlet and outlet culture medium ports to stabilize and seal the tubes (highlighted with gray in Figs. 1c, 2a). For long-term culture, the well plate and the external medium reservoirs were placed inside a conventional CO_2_ incubator while the peristaltic pump was located outside. The square PDMS cover, illustrated in Fig. 1c, was used to isolate and protect the cell culture areas from the incubator during culture. Real-time spatiotemporal imaging of constitutive cells in the sinusoid and stroma chambers were achieved using an inverted microscope (Nikon Ti-E) equipped with an automated stage housed in an incubator with CO_2_, humidity, and temperature controls.

### Tissue construction in sinusoid and stroma chambers

Detailed procedures used for cell preparation and seeding are provided in Methods. In brief, EA.hy926 cell line (CRL-2922, ATCC), derived from human umbilical vein ECs, were used since human EC cell lines derived from BM are not available to our best knowledge and obtaining human primary BM ECs was beyond the scope of this model development study. ECs were prelabelled with CellTracker Green CMFDA Dye (C2925, ThermoFisher). HS-5 human stromal cell line (CRL-11882, ATCC) derived from adult human BM stroma and widely used as feeder cells in ex vivo BM cultures^27^, was used as a surrogate of BMSCs. BMSCs were prelabelled with CellTracker Red CMTPX Dye (C34552, ThermoFisher). The sinusoid chamber was seeded with ECs at the cell density of 5 × 10^6^ cells/mL. The stroma chamber was seeded with 60 μL BMSC/collagen mixture. 2 mg/mL of collagen was used as an optimum concentration that provides stiffness to support stromal cells during 3D culture. The BMSC density in the collagen mixture was 0.5 × 10^6^ cells/mL.

The seeded ECs and BMSCs were dynamically cultured for up to 24 days while flowing culture medium through the sinusoid chamber at a volumetric flow rate of 8.98 µL/min. The culture medium above the BMSC chamber was replenished every 3 days. After culture, cells were fixed and stained with primary mouse anti-human antibody, CD31 (platelet endothelial cell adhesion molecule-1, JC70, sc-53411, Santa Cruz Biotechnology) and DAPI (D9542, Sigma-Aldrich). CD31 was used as a maker for ECs. Also, we used CD31 distribution as an indicator for the formation of intercellular junctions since: (1) CD31 is known to redistribute at cell borders during junction formation^28^, (2) highly enriched CD31 at EC junctions can be related to vascular integrity^29^, (3) the loss of CD31 expression appears to induce the contraction of ECs and vascular barrier breach^30^. DAPI was used to stain cell nuclei. The stained cells were analyzed using a Zeiss LSM 880 series laser scanning confocal microscope. Some tissue samples were fixed and dehydrated by serial ethanol washing and critical point drying for examination using a Zeiss Auriga Crossbeam 40 scanning electron microscope (SEM). Detailed procedures used for cell fixing, staining, and imaging are provided in Methods.

After 4 h of static culture, ECs formed a confluent layer on the sinusoid chamber walls (Fig. S2c). As shown in Figs. S2a and S2b, we used brightfield optical imaging to characterize the pore structure of the PETE membrane and their effects on the development of ECs. Fig. S2a shows ~ 10 μm pores unevenly distributed on the membrane surface prior to EC seeding. Fig. S2b shows that most pores were covered by ECs after static culture for 4 h.

**Figure 4:**
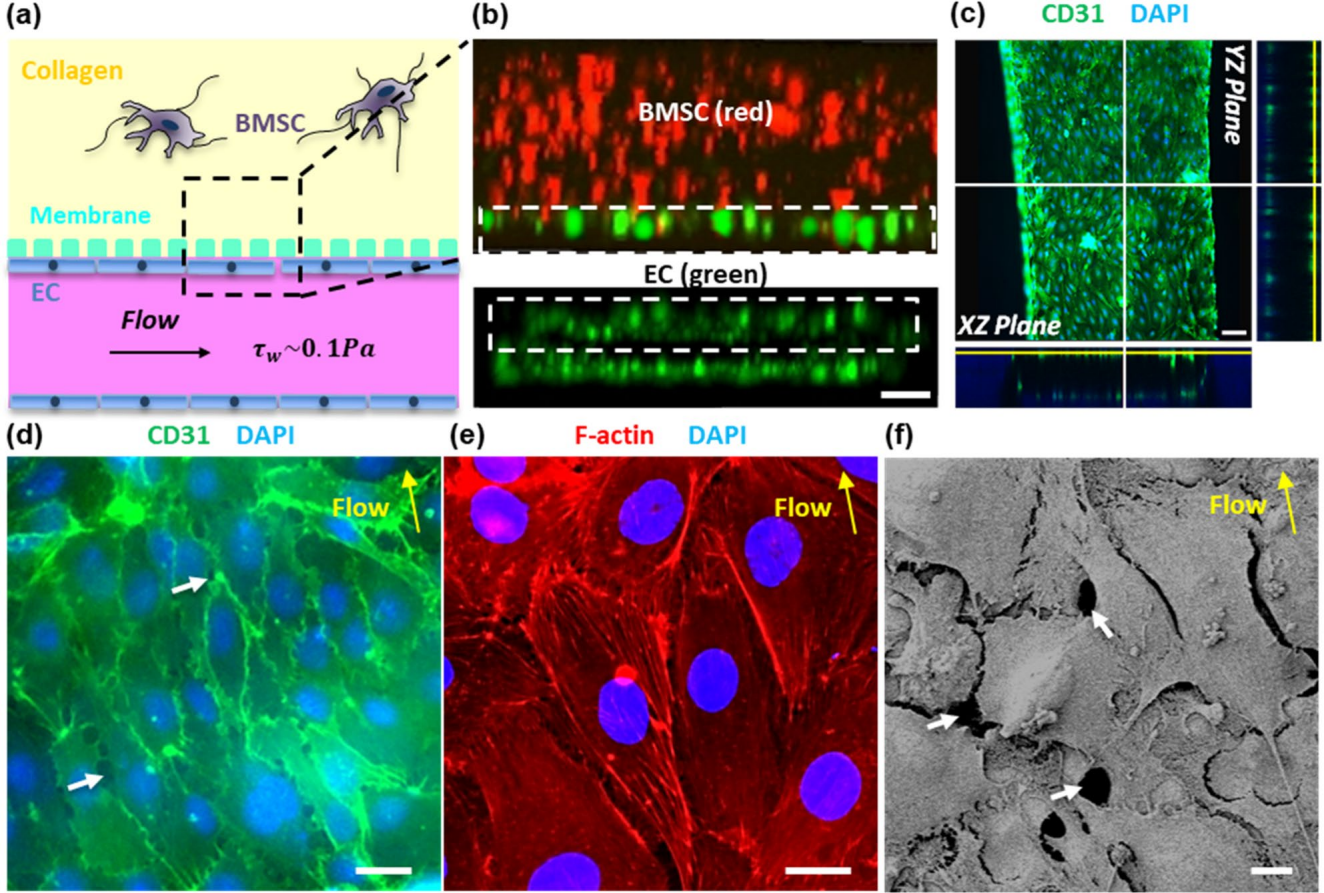
ECs and BMSCs cultured in the sinusoid and stroma chambers. (**a**) Schematic of the integrated sinusoid/stroma chambers illustrated for spatial reference. (**b**) Confocal cross-sectional imaging of ECs (green, CMFDA) and BMSCs (red, CMTPX) cultured for 12 h in the sinusoid chamber (bottom) and the stroma chamber (top). ECs labeled with CellTracker Green CMFDA and BMSCs labeled with CellTracker Red CMTPX Dye. Scale bar = 100 μm. (**c**) Confocal sections of ECs cultured in the sinusoid chamber for 24 days in the z-projection direction. Green (CD31) and Blue (DAPI). Scale bar = 100 μm. (**d**) Enlarged view of ECs from (**c**). Green (CD31) and Blue (DAPI). Scale bar = 20 μm. (**e**) ECs stained with F-actin (red), showing F-actin fibers aligned along the direction of flow after 24 days of culture. Scale bar = 10 μm. (**f**) SEM image showing junctions and contacts formed between ECs after 24 days of culture. Scale bar = 10 μm. The white arrows in (**d**) and (**f**) point to open gaps between ECs. The yellow arrows in (**d**) through (**f**) indicate the direction of flow in the sinusoid chamber.

Figure 4a shows the schematic configuration of the integrated sinusoid/stroma chambers and Fig. 4b shows the overall distribution of ECs and BMSCs cultured for 12 h in the sinusoid and stroma chambers. The cross-sectional view of the sinusoid chamber (the bottom image in Fig. 4b) shows that ECs covered all the chamber walls, forming a rectangular lumen-like structure with 80–100 μm in height and 800 μm in width. The top image in Fig. 4b shows that BMSCs were uniformly distributed in the collagen matrix of the stroma chamber. These results indicate that an endothelial lumen structure could be formed in the sinusoid chamber within 12 h while BMSCs being uniformly dispersed in collagen and co-cultured in the stroma chamber.

After 24 days of culture, the endothelial lumen structure was stained with CD31 which was strongly expressed at EC junctions (Fig. 4c,d). The localized expression of CD31 indicated that the endothelial phenotype was maintained along with the formation of intercellular junctions^31^. The effects of flow-induced shear stress were evident on the morphological alignment of ECs (Fig. 4d) and the re-arranged cortical organization of F-actin (Fig. 4e) along the direction of flow. The effects of increasing shear stress from 0.01 to 0.1 Pa on the increased elongation, stronger CD31 expression, and more oriented F-actin filaments of ECs were also evident as described in Supplementary Information 3 and shown in Fig. S4. Small pores of about 1 to 8 μm were observed at some EC junctions, as indicated by the white arrows in Fig. 4d and from Supplementary Video 1. Especially, the SEM image (Fig. 4f) shows that ECs were well connected to each other, despite the formation of small pores. In this image, membrane holes were not visible, as the membrane surface was mostly covered by the EC layer.

Figures S3a and S3b show BMSCs cultured for 24 days with collagen in the stroma chamber. In comparison to BMSCs cultured for 12 h (Fig. 4b), it was apparent that BMSCs were uniformly distributed in the stroma chamber during the 24-day period. The SEM images in Figs. S3c and S3d show the detailed morphological features of BMSCs developed within the collagen matrix. BMSCs were elongated with many dendrite-like protrusions through the collagen fibers. In the absence of any detectable migration of BMSCs towards the ECs, it appeared that these cells did not physically interact.

### Barrier function of endothelium

CD31 expression is associated with the restrictive barrier function of endothelium in vivo^31^. The barrier function of the endothelium was estimated by measuring the permeability ($P_{D}$) of a fluorescein isothiocyanate-dextran solution (70 kDa, 20 μg/mL in PBS, Sigma-Aldrich) through the sinusoid chamber (detailed procedures provided in Methods and Supplementary Information 4). Briefly, as shown in Fig. S5, fluorescent images of dextran diffusing from the sinusoid to the stroma chamber were acquired at 15 s/frame for 180 s using the Nikon Ti-E microscope. Videos were taken at 0, 2, 5, 10, 15, 20, and 30 min to follow the dextran distribution. $P_{D}$ was calculated using the dextran diffusion model previously developed by William et al.^32^.

**Figure 5:**
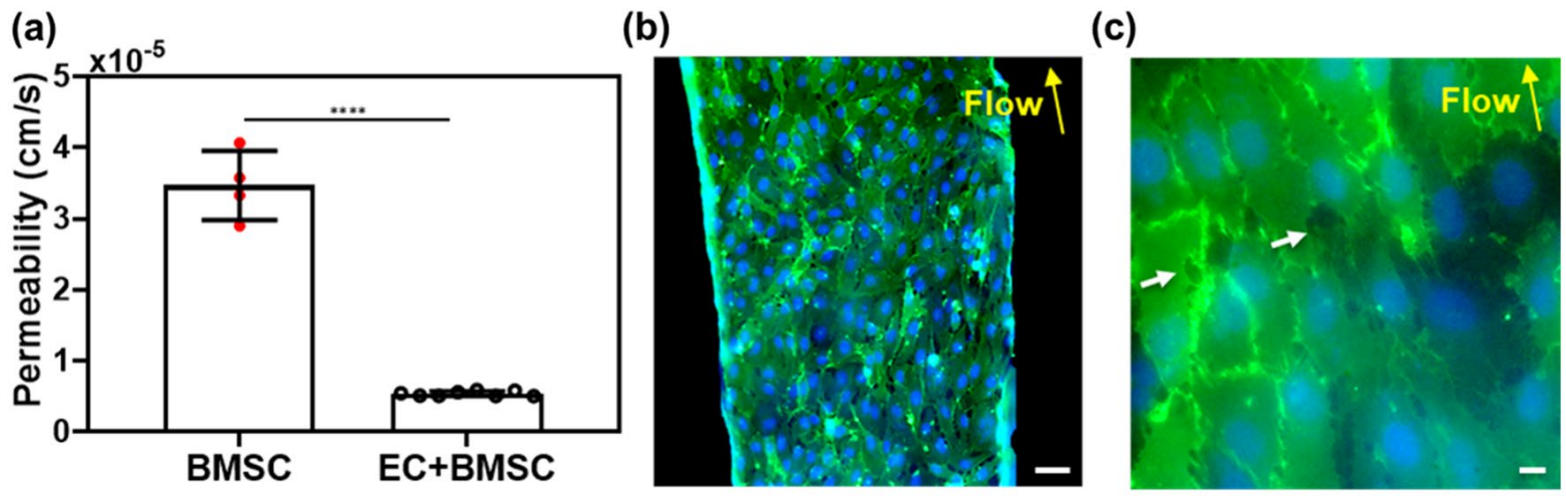
Permeability (${P}_{D}$) of 70 kDa FITC-dextran through the endothelium constructed between the sinusoid and stroma chambers. ECs were dynamically cultured on the membrane surface of the sinusoid chamber for 30 h. (**a**) ${P}_{D}$ measured in 6 culture samples per condition (n = 6, mean ± SD). ****P < 0.0001 determined by t-test. (**b**) and (**c**) Confocal fluorescence images of the endothelium formed after dynamic culture for 30 h. CD31 (green) and DAPI (blue). The white arrows indicate small pores located at some EC junctions. Scale bars: 100 μm in (**b**) and 10 μm in (**c**). The yellow arrows in (**b**) and (**c**) indicate the direction of flow in the sinusoid chamber.

$P_{D}$ through the PETE membrane after BMSCs were cultured for 30 h in the collagen matrix, but without ECs in the sinusoid chamber (i.e., “BMSC” in Fig. 5a) was measured to be ~ 3.5 ± 0.5 × 10^–5^ cm/s. When ECs were placed and cultured in the sinusoid chamber (i.e., “EC + BMSC” in Fig. 5a), $P_{D}$ was significantly decreased to about 5.8 ± 0.2 × 10^–6^ cm/s; suggesting that the EC layer significantly increased resistance to the diffusion of dextran. In separate experiments using the same culture conditions, ECs were characterized by staining with CD31 and DAPI. Figure 5b shows that ECs formed a uniform endothelium layer on the PETE membrane surface. Figure 5c shows well connected junctions between ECs with the occasional presence of small pores of 1.5 to 10 μm with an average pore size of 3.4 μm.

### CXCL12-induced egression of MM cells

In order to demonstrate that the device can be used to study the trafficking of MM cells through the sinusoidal niche, we evaluated the effects of CXCL12 on meditating the egression of MM cells from the BM stroma chamber. It has been reported that MM cells express the chemokine receptor CXCR4 and are therefore attracted to CXCL12^+^ cells in the BM^16^. CXCL12 can also induce cytoskeletal rearrangement, pseudopodia formation, and internalization of the CXCR4 receptor in MM cells^16,33^. CXCL12 is also known to upregulate VLA-4–mediated MM cell adhesion to fibronectin and VCAM-1^16,34^ and increases invasion and matrix metalloproteinases (MMP) secretion^16,35^. In this study, we used the human MM.1S cell line which has been widely used to study MM and the development of drug resistance^15,36^.

**Figure 6:**
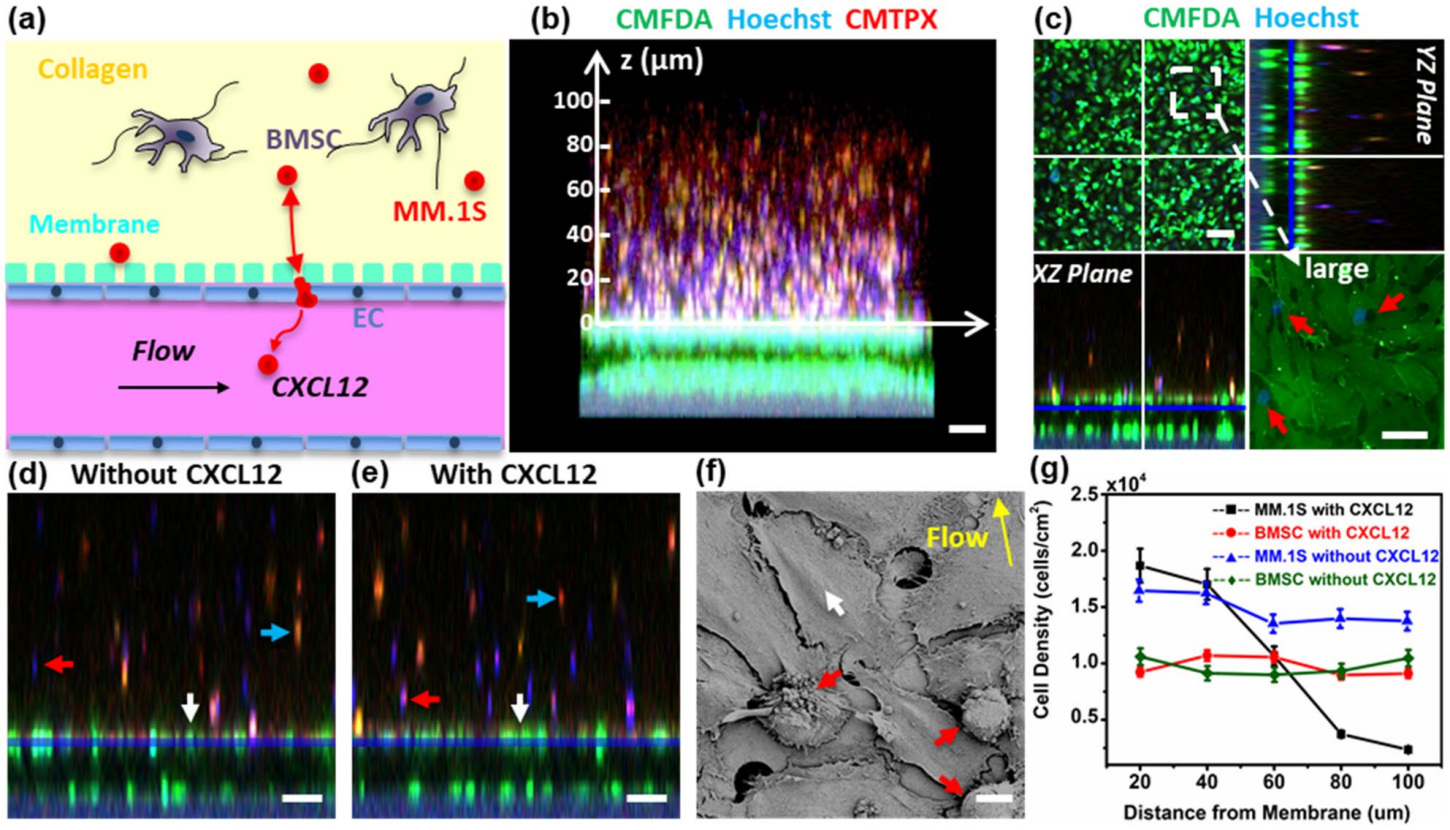
CXCL12-meditated egression of MM.1S cancer cells from the BM stroma chamber. (**a**) Schematic representation of cells inside the device. (**b**) Confocal cross-sectional view of the device after adding CXCL12-containing medium in the sinusoid chamber for 4 h; ECs (CMFDA green), BMSCs (CMTPX red), MM.1S cells (Hoechst blue). Scale bar = 200 µm. (**c**) Z-stack projection of confocal fluorescence images of MM.1S migration after adding CXCL12. Scale bar of z-projection = 100 µm. Scale bar in the larger image = 20 µm. (**d**) and (**e**) Cross-sectional confocal fluorescence views with and without CXCL12, respectively. Scale bar = 100 µm. (**f**) SEM image of the endothelium infiltrated with MM.1S cells. Scale bar = 10 µm. (**g**) Number density of Hoechst-stained MM.1S cells was manually counted and averaged at six different locations (n = 6) in the z-direction of (**b**). In (**c**)–(**f**), red arrows indicate MM.1S cells, white arrows indicate ECs, blue arrows indicate BMSCs, and yellow arrow indicates flow direction.

Figure 6a illustrates the experimental configuration used to induce the egression of MM1.S cells from the stroma chamber to the sinusoid chamber by adding CXCL12 (R&D Systems) to the culture medium flow in the sinusoid chamber (640 ng/mL). ECs, BMSCs, and MM.1S cells were pre-labeled with CMFDA (green), CMTPX (red), and Hoechst (blue), respectively. MM.1S cells (2 × 10^6^ cells/mL) and BMSCs (0.5 × 10^6^ cells/mL) were thoroughly mixed into collagen before placing the mixture in the stroma chamber. The cells in the device were cultured using the 1:1 mixture of the DMEM and RPMI complete cell culture media. CXCL12 was added in the common culture medium fed into the sinusoid chamber after ECs, BMSCs, and MM.1S were cultured in the device for 26 h. Fluorescence images and videos were taken in situ using the confocal and fluorescence microscopy for the following 4 h (Supplementary Information 5). To help maintain the CXCL12 gradient, the culture medium in the culture medium reservoir was replenished every 10 min during the 4-h period.

Figure 6b shows the migration of MM.1S cells from the stroma chamber to the sinusoid chamber as evident from the higher density of Hoechst-stained MM.1S cells toward the endothelium. In Fig. 6d,e, the cross-sectional confocal fluorescence views imaged without and with adding CXCL12, respectively, are compared. MM.1S cells were evenly distributed in the stroma chamber in the absence of CXCL12 (Fig. 6d) whereas more MM.1S cells were observed at the stroma/sinusoid interface with CXCL12 (Fig. 6e). In contrast, the spatial distribution of BMSCs (red cells) was not affected by adding CXCL12. Figure 6g shows the migration data quantified by counting the number of MM.1S cells as a function of z-axis positions specified in Fig. 6b. The cell density of MM.1S cells decreased in the axial direction of the stroma chamber toward the sinusoid chamber, as expected from their migration toward the high concentration of CXCL12 in the sinusoid chamber. In contrast, the density of BMSCs did not change along the axial direction with or without CXCL12.

The presence of migrating MM.1S cells (indicated by the red arrows in Fig. 6c) in the endothelium was observed during the z-stack confocal fluorescence analysis. From Fig. 6c, the average pore size at junction gaps was measured to be 2 to 20 µm. Figure 6f shows a representative SEM image of the endothelium with small junction gaps as well as the presence of MM.1S cells identified by their round morphology. Furthermore, time-lapse imaging confirmed the CXCL12-induced migration of MM.1S cells toward the endothelium (Supplementary Videos 2 through 6). Fig. S6 shows BMSCs and MM.1S cultured in the stroma chamber at 4 h after adding CXCL12 in the sinusoid chamber. No close contacts between MM.1S cells and BMSCs were detected. Taken together, these results suggested that MM.1S cells migrated through the endothelium in response to CXCL12 while BMSCs did not. We further evaluated the CXCL12 production by BMSCs and ECs by ELISA. As detailed in Supplementary Information 5, BMSCs and ECs produced very low levels of CXCL12 (below 100 pg/mL), supporting that: (1) MM.1S cells did not migrate to BMSCs and ECs via CXCL12/CXCR4 axis and (2) the migration of MM.1S cells was induced by the addition of CXCL12 in the sinusoid chamber.

**Figure 7:**
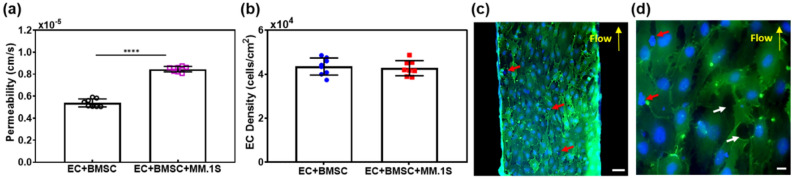
Effects of CXCL12-meditated egression of MM.1S cancer cells on dextran permeability, EC viability, and EC junction pore density. (**a**) ${P}_{D}$ of 70 kDa FITC-dextran between ECs and BMSCs cultured for 26 h followed by the addition of CXCL12 for 4 h (EC + BMSC) versus ECs, BMSCs, and MM.1S cells cultured for 26 h and then CXCL12 added for 4 h (EC + BMSC + MM.1S). The mean ± SD of six samples per condition were analyzed. ****P < 0.0001 determined by t-test. (**b**) Viability of ECs measured for “EC + BMSC” versus “EC + BMSC + MM.1S” (8 images analyzed per condition, mean ± SD). (**c**) and (**d**) Confocal fluorescence images of the endothelium, showing MM.1S located at EC junction pores in the presence of CXCL12. The white arrows indicate junction pores, the red arrows indicate MM.1S cells, and yellow arrows indicate flow direction. Scale bars: 100 μm in (**c**) and 10 μm in (**d**).

The effects of CXCL12-mediated MM.1S cell migration on the barrier function of ECs were evaluated by measuring $P_{D}$ and characterizing the morphological features of these cells. As described in the previous section, CXCL12 was introduced for 4 h in the sinusoid chamber after BMSCs. Figure 7a shows that $P_{D}$ significantly increased from 5.8 ± 0.2 × 10^–6^ to 9.3 ± 0.1 × 10^–6^ cm/s when MM.1S cells were present and their migration was induced. As indicated by the red arrows in Fig. 7c,d, the presence of MM.1S cells was observed throughout the endothelium layer, confirming migration. As detailed in the Methods section and shown in Fig. 6b, we pre-labeled ECs with CMFDA (green), MM.1S cells with Hoechst (blue), and BMSCs with CMTPX (red). For the confocal images in Fig. 7c,d, the cells were further fixed and stained with CD31 (green) and DAPI (blue). In Fig. 7d, round cells stained with blue only (both Hoechst and DAPI) were identified as transmigrated MM.1S cells. Elongated green cells (CMFDA) with stronger green intensity at cell junctions (CD31) and blue nuclei (DAPI) were identified as ECs.

As indicated by the white arrows in Fig. 7d, junction gaps of 2 to 20 μm formed between ECs. These gaps were substantially larger than those of 1.5 to 10 μm formed in the absence of MM.1S cells (Fig. 5c). However, the density of ECs was not significantly influenced by the presence and migration of MM.1S cells (Fig. 7b), suggesting that the viability of ECs was not compromised by MM.1S migration. These results suggested that the migration of MM.1S cells was accompanied by openings in the EC junction pores and thus an increase in the $P_{D}$ of dextran through the endothelium layer without affecting the viability of ECs.

## Discussion

In this study, we developed a microfluidic device specifically designed to recapitulate the BM-embedded sinusoidal niche, which provides circulation to the BM and facilitates the migration of cancer cells that reside and/or metastasize to the BM microenvironments. We show that an endothelial lumen structure could be formed in the sinusoid chamber within 4 h while BMSCs being uniformly dispersed in collagen and co-cultured in the stroma chamber (Fig. 4b). During subsequent dynamic culture at *τ*_*w*_ ~0.1 Pa for 24 days, the endothelial lumen structure was developed with: (1) the formation of intercellular junctions as evidenced from strong localized CD31 expression (Fig. 4c,d), (2) the morphological alignment of ECs (Fig. 4d), and (3) the rearranged cortical organization of F-actin (Fig. 4e) along the direction of the flow. These shear-induced morphological developments were consistent with the previously reported observations^32,37^. In particular, William^32^ observed significant shear-induced alignment and rearrangement effects at *τ*_*w*_ up to 0.5 Pa.

BMSCs were distributed in the BM stroma chamber (Figs. S3a and S3b), became elongated with extended protrusions (Figs. S3c and S3d), and did not migrate towards the ECs. The morphological developments were similar to those observed from the 3D culture of HS-5 cells. The lack of migration of HS-5 cells towards ECs was consistent with previous observations by Surya et al.^27^.

The barrier function of the endothelium was quantitatively assessed by measuring the intensity of 70 kDa FITC-dextran in the collagen-filled stroma chamber (Fig. 5a). $P_{D}$ = 5.8 ± 0.2 × 10^–6^ cm/s, which was close to that reported for endothelial monolayers formed in vitro under *τ*_*w*_ =0.2 Pa (4.1 ± 0.5 × 10^−6^ cm/s for 70 kDa dextran)^37^. Importantly, our experimental value was higher, but within the order of magnitude for $P_{D}$ (1.2 ± 0.2 × 10^–6^ cm/s) of dextran measured in vivo using intravital microscopy in sinusoidal microvessels within the BM^24^. Morphologically, the presence of EC pores of 1.5–10 μm was observed at EC junctions (Fig. 5c). In comparison, BM sinusoidal endothelial cells formed a highly fenestrated lumen with the average size of openings of 1.2–2 μm^23,38^. The porous structure is important since previous studies^21,39^ suggested that the transmigration of BM cells through the sinusoidal endothelium occurs mainly through these small pores located at EC junctions. The comparison of our results with in vivo observations indicated that the porous and leaky barrier function of BM sinusoidal microvessels could also be recapitulated in the device.

The live imaging, immunofluorescence, and SEM results in Fig. 6 show that CXCL12 induced the egression of MM.1S cells from the stroma chamber. These results also suggested that MM.1S cells transmigrated through openings at EC junctions. In contrast, BMSCs did not migrate and MM.1S cells only did so in the presence of CXCL12. Cytokine analysis (Supplementary Information 5) showed that BMSCs and ECs do not produce CXCL12 at significant levels. Taken together, the results support that the migration was specifically induced by adding CXCL12 in the sinusoid chamber.

Interestingly, the presence and transmigration of MM.1S cells caused the widening of EC junction pores (Fig. 5c vs. Fig. 7d) and significantly increased the $P_{D}$ of dextran (Fig. 7a), but did not compromise the viability of ECs (Fig. 7b). Also, it appeared that ECs became more disorganized and loosely connected (Fig. 5b,c vs. Fig. 7c,d). These morphological changes of ECs appear to mimic angiogenic abnormalities observed in sinusoidal ECs during active MM progression^40,41^. It is known^23^ that these abnormalities are usually caused by avid binding and uptake of cationic liposomes to ECs and the expression of integrins, growth factors, and receptors that differ from those of normal endothelium. We hypothesize that MM cells produce vascular endothelial growth factor (VEGF), which is known to promote the development of angiogenic abnormalities in ECs and makes endothelium to become more porous and leakier^42^. Both MM.1S and RPMI-8226 (also a human MM cell line) cells are known to express CXCR4 and VEGF^43^. As mentioned earlier, CXCL12 is also known to upregulate the secretion of MMP and thus increases for MM cell invasion^35^. These mechanisms may explain our observations on the morphological changes of ECs induced by MM.1S cells, and will be investigated and ascertained in follow-up studies.

While prior studies have focused on angiogenesis and MM growth in the BM^44,45^, we could not find any specific work on the effects of MM cell trafficking on the barrier function of ECs. In this regard, our results invite a mechanistic question as to whether: (1) MM cell trafficking relies on the ability of these cells to penetrate and deform the cytoplasm of ECs and/or (2) ECs contribute to the trafficking process, e.g., by discharging lysosomal vesicles and segmental destabilization^21,46^. It will be important to follow the phenotypic changes that MM cells undergo during egression in comparison to in vivo observations.

From the device perspective, the overall results support that the microfluidic device could be used to mimic several physiological features of the BM sinusoidal niche: (1) sinusoidal blood circulation in the BM, (2) the sinusoidal endothelium of the BM, and (3) BM stromal compartment. The recapitulated multicellular tissue compartments were utilized to demonstrate the device’s capability in examining the effects of MM cell egression on the barrier function of endothelium. For future work, we can place a layer of osteoblasts on the top of the stroma chamber to mimic the endosteal niche and to integrate with the two other major microenvironments of the BM (i.e., sinusoidal and stromal). ECs and BMSCs isolated directly from BM samples can be used instead of cell lines, and patient-derived CD138^+^ MM cells can also be incorporated for clinically relevant studies. With these advances, we anticipate that our microfluidic device will enable the systematic study of how specific niches within the BM contribute to the survival, drug resistance, and progression of MM.

Beyond its application to unravel the biology of MM and its interdependence with the BM niche, the device can be manufactured test personalized approaches that would decrease MM cell migration and spreading. In particular, our prior work in the role of osteoblasts in MM survival had yielded important insights as to potential targetable proteins (e.g., N-cadherin) that could decrease the pernicious interaction of MM cells^5^. Likewise, the device could be used to screen antibodies and small molecules that are designed to mitigate the interaction of MM cells with ECs and other supportive stromal elements (e.g., IL27^47^, JAM-A^48^). Alone or in combination with standard of care therapies, the device could enable the discovery of new and repurposed molecules capable of disrupting the MM-microenvironment interactions known to confer drug resistance. For these envisioned studies, drugs can be added to the vascular compartment to mimic their delivery mechanisms. We note that we previously developed and demonstrated methodologies to perform cytotoxic assays and cell viability using fluorescence microscopy in our microfluidic devices^5,7^.

While in vitro 3D BM tissue models have become biologically more functional^49–52^, they are not yet sufficiently complex to capture and resolve the spatiotemporal microenvironments of the cancer niches. In this regard, our device uniquely integrates and captures the sinusoidal and stromal compartments in a spatially resolved manner while mimicking the physiological *τ*_*w*_ of sinusoidal blood flow (10^–1^ Pa). This sinusoidal flow feature provides an integral element for mimicking the trafficking of circulating cancer cells. Therefore, the utility of the device can be further extended to create systemic models of bone metastasis of MM, breast cancer^53^, and prostate cancer^54^.

In conclusion, the microfluidic device was designed to mimic: (1) sinusoidal blood circulation in the BM, (2) the sinusoidal endothelium of the BM, and (3) the BM stroma. The utility of the device was demonstrated by spatiotemporally characterizing the CXCL12-mediated egression of MM cells from the BM stroma and its effects on the endothelial barrier function. We anticipate that the device can be further developed and used to study the spatiotemporal interactions of cancer cells during their trafficking through the BM sinusoidal niche.

## Methods

### Device fabrication

The microfluidic device (Figs. 3 and S1) was fabricated using previously reported techniques^5,7,25^. Briefly, the device was assembled using a bottomless 96-well plate (Grenier bio one), polydimethylsiloxane (PDMS, Sylgard 184, Dow Corning) layers, pressure-sensitive adhesive (PSA, Arcare 90,106, Adhesive Research) layers, a transparent plastic coverslip (S17525B, Fisher Scientific), and a transparent polyester (PETE) membrane layer (Sterlitech PET10025100, 10 μm pores, and 9 μm thickness). According to the supplier’s specifications, the membrane contained the surface density of 10^5^ pores/cm^2^ and the overall open surface area of 7.9%. The PSA and PDMS layers were cut and patterned using a digital craft cutter (Silhouette CAMEO) with a spatial resolution of 500 µm. As we recently published^7^, digital cutting enabled entire device fabrication to take less than 2 h, including PDMS preparation. For more details of the device fabrication procedures are provided in Supplementary Information 1.

### Device sterilization and preconditioning

After UV sterilization for 2 h, the sinusoid chamber was washed three times with autoclaved PBS and dried for 1 h in the laminar flow hood. The stroma chamber was washed 3 × with PBS and dried for 1 h in the hood. Subsequently, the sinusoid chamber was coated with 100 μg/mL type I collagen (5056, Advanced BioMatrix) for 2 h at room temperature to increase the adhesion of ECs. The device was flipped after slowly injecting the collagen solution, washed 3 × with PBS, and dried in the hood.

### Cell preparation

Prior to use in the microfluidic device, EA.hy926 endothelial cells, passage 12–18, were cultured in growth media Dulbecco's Modified Eagle's Medium (DMEM, ATCC) supplemented with 10% fetal bovine serum (FBS, ATCC) until they reached 80% confluency. ECs were prelabelled with CellTracker Green CMFDA Dye (5 µM, C2925, ThermoFisher) as per the manufacturer’s instructions and resuspended at a cell density of 5 × 10^6^ cells/mL. BMSCs were cultured in DMEM supplemented with 10% FBS and 1% penicillin–streptomycin sulfate (Corning) until they reached 70%confluency followed by prelabelling with CellTracker Red CMTPX Dye (5 µM, C34552, ThermoFisher). The prelabelled BMSCs were resuspended in 2 mg/mL collagen for seeding at the cell density of 0.5 × 10^6^ cells/mL. Type I rat tail collagen (5153, Advanced BioMatrix) was used to disperse and culture BMSCs. The human MM.1S cell line (CRL-2974, ATCC) established from the peripheral blood of a MM patient was expanded at 37 °C, 5% CO_2_ in RPMI-1640 media supplemented with 10% FBS, 2 mM of L-glutamine, 100 U/m penicillin, and 100 mg/mL streptomycin.

### Cell seeding

After the sinusoid chamber was slowly infiltrated with ECs, the device was flipped for 1 min. A bright-field microscope was used to quickly check that the cells were flowing through the chamber and forming a homogeneous layer. The seeding step was completed within 15 min to minimize the time that the cells were in a suspended state. After seeding the ECs, the culture medium was added to both the sinusoid and stroma chambers and the device was flipped for 20 min at 37 °C in the incubator to allow cell adhesion to all the walls of the sinusoid chamber. Culture medium (50 μL) was used to wash away nonadherent ECs. Once the sinusoid chamber was seeded, the medium in the stroma chamber was removed. The stroma chamber was then seeded with a 60 μL mixture of BMSCs and neutralized collagen solution (2 mg/mL). This seeding step was quickly performed to avoid the gelation of collagen. All steps associated with handling collagen was performed on ice. The device was flipped for 1 min to provide more time for ECs to adhere to the membrane. The entire seeding procedures for the sinusoid and stroma chambers seeding took about 4 h.

### Cell culture

The device was placed in the incubator for 30 min at 37 °C and 5% CO_2_ to allow collagen gelation. Subsequently, 200–250 μL of culture medium was added to fill the stroma chamber and the culture medium reservoir. The PDMS cap was placed to seal the stroma chamber. The device was connected to the culture medium circulation system to begin dynamic culture. The seeded ECs and BMSCs were dynamically cultured in DMEM medium supplemented with 10% FBS, and at various time points, ECs and BMSCs were imaged using the Nikon Ti-E fluorescence microscope.

### Immunofluorescence staining

After culture, in situ fixation and immunofluorescence staining were carried out. The cells in the sinusoid chamber and the stroma chamber were fixed in situ using 4% paraformaldehyde (PFA, J61899-AP, ThermoFisher) solution for 30 min and washed 3 × with PBS. The device was incubated for 30 min in a blocking solution consisting of 2% bovine serum albumin (BSA, A2153, Sigma-Aldrich) and 0.5% Triton-X 100 (X-100, Sigma-Aldrich) for membrane permeabilization. Primary mouse anti-human antibody CD31 (platelet endothelial cell adhesion molecule-1, JC70, sc-53411, Santa Cruz Biotechnology) was added to the sinusoid chamber and incubated overnight at 4 °C. The chamber was washed 3 × with PBS. The secondary antibody goat anti-mouse IgG H&L Alexa Fluor 488 (Abcam, ab150113) and nuclear counterstain DAPI were then added to the sinusoid chamber for 2 h and washed 3x.

### Confocal microscopy

Immunofluorescence z-stack images (1 μm step size) of the sinusoid chamber were taken with a Zeiss LSM 880 series laser scanning confocal microscope with 10 x, 20 x, 40 x (water immersion) and 63x (oil immersion) objectives. Z-projections, cross sections, and 3D reconstructions of confocal images were generated using ZEN3.1 (blue edition) software.

### Scanning electron microscopy (SEM)

The cells in the device were fixed in situ using 25% glutaraldehyde overnight before dehydrated in serial ethanol washes (30%, 50%, 70%, 85%, 95%, and 100% ethanol). The device was placed in 100% ethanol for 2 days, while replenishing ethanol every 12 h. The device was disassembled after overnight freezing in a − 80 °C freezer. The plastic coverslip layer was removed to expose the endothelium formed on the membrane for SEM specimens preparation. After critical point drying (Tousimis, SamDri-780), the tissue samples were sputter-coated with gold and analyzed by a Zeiss Auriga Crossbeam 40 scanning electron microscope.

### Permeability measurements

A 10 × magnification objective was used to image the cross-section of the entire stroma and sinusoid chambers (Fig. S5a). The image sequences were analyzed with Image J and MATLAB R2020b using the dextran diffusion model previously developed by William et al.^32,55,56^. The diffusion model assumes that the intensity of fluorescence is proportional to the number of dextran molecules in the solution. Also, the model assumes that $n_{Stroma \ll } n_{Sinusoid}$ for initial flux where $n_{Stroma}$ is the number of dextran molecules in the stroma chamber and $n_{Sinusoid}$ is the number of dextran molecules in the sinusoid chamber^32,56,57^. Based on the assumptions, the permeability of dextran, $P_{D}$, can be related to fluorescence intensity using the following equation:^32,37,56^

1 $$P_{D} = \left( {\frac{h}{{I_{Sinusoid} }}} \right)\left( {\frac{{dI_{Stroma} }}{dt}} \right)$$

Details of the Permeability measurements procedures can be found in Supplementary Information 4.

## Supplementary Information

Supplementary Legends.

Supplementary Information 1.

Supplementary Video 1.

Supplementary Video 2.

Supplementary Video 3.

Supplementary Video 4.

Supplementary Video 5.

Supplementary Video 6.

## Data Availability

All data supporting the findings of this study are available within the article and its Supplementary Information Files.
