# Supplementary material for: Microfluidic device engineered to study the trafficking of multiple myeloma cancer cells through the sinusoidal niche of bone marrow
Source: Sci Rep. 2022 Jan 27;12:1439. doi: 10.1038/s41598-022-05520-4 (PMC8795452; doi:10.1038/s41598-022-05520-4)
Supplement: Supplementary file 1 — Supplementary Legends. [file 41598_2022_5520_MOESM1_ESM.docx]

**Supplementary Video 1.** Rotating view of the confocal 3D image of the lumen morphology in the sinusoid chamber after microfluidic culture for 24 days. EA.hy926 cells stained by CD31 (green) and DAPI (blue).

**Supplementary Video 2.** Time-lapse video of the migration behavior of MM.1S cells through the EA. hy926 cell layer formed in the sinusoid chamber in 4 h. EA.hy926 cells were prelabeled with CMFDA (green). HS-5 stromal cells in the stroma chamber were pre-labeled with CMTPX (red). MM.1S cells were not stained (i.e., no color). The right side area of the yellow dash line represents the top view looking from the stroma chamber and into the sinusoid chamber. The red arrows indicate migrating MM.1S cells.

**Supplementary Video 3.** Enlarged time-lapse video of a migrating MM.1S cell in Area (i) indicated in Supplementary Video 2.

**Supplementary Video 4.** Enlarged time-lapse video of a transmigrating MM.1S cell through a membrane hole in Area (ii) indicated in Supplementary Video 2.

**Supplementary Video 5.** Enlarged time-lapse video of a transmigrating MM.1S cell through a membrane hole in Area (iii) indicated in Supplementary Video 2.

**Supplementary Video 6.** Enlarged time-lapse video of a transmigrating MM.1S cell through a membrane hole in Area (iv) indicated in Supplementary Video 2.
