# Supplementary material for: Microfluidic device engineered to study the trafficking of multiple myeloma cancer cells through the sinusoidal niche of bone marrow
Source: Sci Rep. 2022 Jan 27;12:1439. doi: 10.1038/s41598-022-05520-4 (PMC8795452; doi:10.1038/s41598-022-05520-4)
Supplement: Supplementary file 2 — Supplementary Information 1. [file 41598_2022_5520_MOESM2_ESM.docx]

**Supplementary Information 1 – Device Fabrication**

The first PSA layer was directly adhered to the coverslip and used to create the sinusoid chamber and the bubble trap (Figs. 3b, 3c and Fig. S1). The transparent polyester (PETE) membrane was digitally cut and placed between the first and second PSA layers. The second PSA layer was used to: (1) place the PETE membrane, (2) control the shape and dimensions of the stroma chamber, and (3) produce the bubble storage area of the bubble trap. The first PDMS layer was used to build the thickness of the stroma chamber to 0.5 mm (Figs. 2b, 3c, and Fig. S1) and to help seal the microfluidic passages to be leak-tight. The third PSA layer was used to pattern and produce the culture medium reservoir and the BMSC seeding ports. The fourth PSA layer was used to adhere the microfluidic device to the bottomless well plate. The second PDMS layer was used to provide the inlet and outlet ports for the sinusoid chamber and the stroma chamber as well as to increase the height of the culture medium reservoir. The PDMS layers were patterned using biopsy punchers (Miltex) of 1.5 mm and 6 mm in diameter. The PDMS layers were plasma-treated prior to bonding to the adjacent PSA layers.


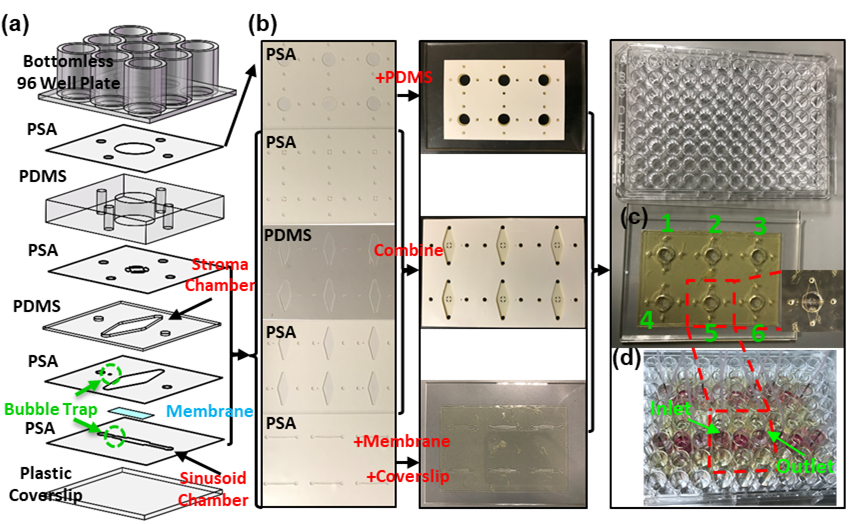


**Figure S1.** Device fabrication. (a) Schematic illustration of the layer-by-layer assembly used to produce the microfluidic device. (b) Actual layers produced and assembled to produce 6 pairs of the sinusoid and stroma chambers on a bottomless 96-well plate. (c) Green number represents 6 pairs of sinusoid and stroma chambers, and enlarge one is one pairs. (d) The real actual device, inlet and outlet arrows are pins that connect to the external tube.

**Supplementary Information 2 – Tissue Construction in Sinusoid and Stroma Chambers**


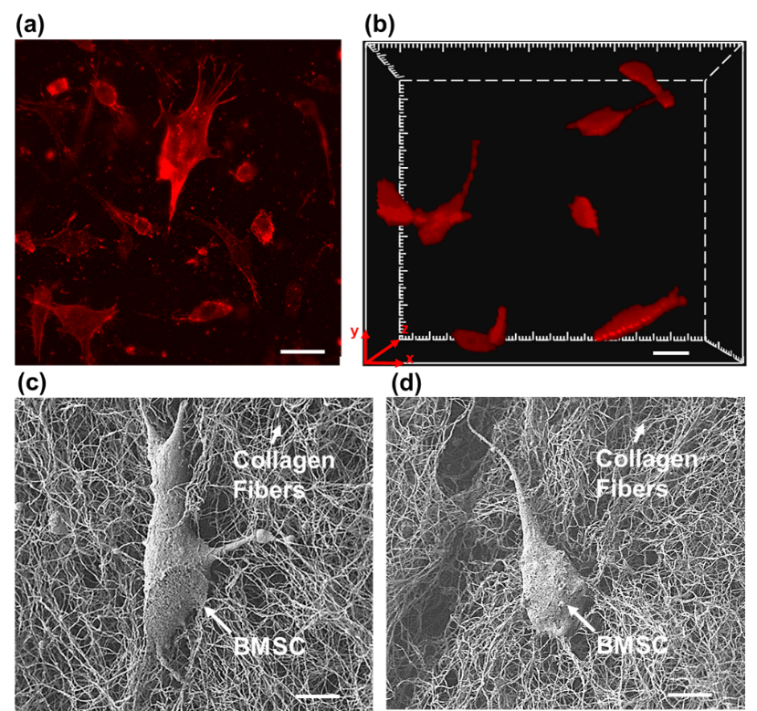


**Figure S3.** BMSCs cultured with collagen for 24 days in the stroma chamber. (a) and (b) 2D and 3D confocal views of BMSCs (Scale bar = 10 μm) and (c) and (d) SEM images (Scale bar = 5 μm).


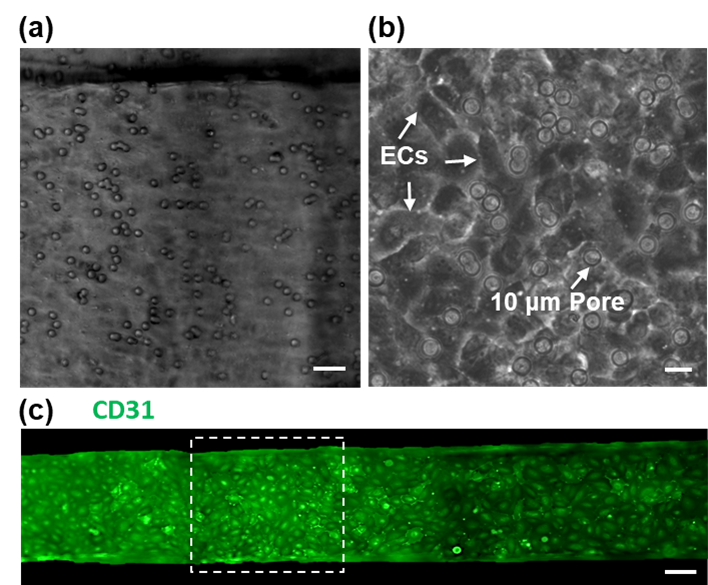


**Figure S2.** (a) Brightfield optical images of PETE membrane, showing the average pore size of 10 μm (Scale bar = 50 μm) prior to culture and (b) Brightfield optical images of ECs formed on the membrane after 4 h. (Scale bar = 20 μm). (c) Confocal fluorescent image of ECs after 4 h, showing the overall distribution of ECs along the length of the sinusoid chamber after 4 h. Green CD31 (Scale bar = 200 μm).

**Supplementary Information 3 – Effect of Shear Stress on Endothelial Cells**

Figure S4 shows the effect of varying shear stress from 0.01 to 0.1 Pa on the elongation of ECs. For this experiment, ECs were seeded at a density of 5x10^6^ cells/mL, were cultured for 72 h under the specified shear stress conditions, and were stained with CD31 and F-actin. The CD31 stain data suggest that the increased shear stress considerably increased: (1) the elongation of ECs along the direction and (2) the expression of CD31, indicating stronger intercellular junction formation. The F-actin stain data also suggest that F-actin filaments become more oriented along the flow direction with the increased shear stress.


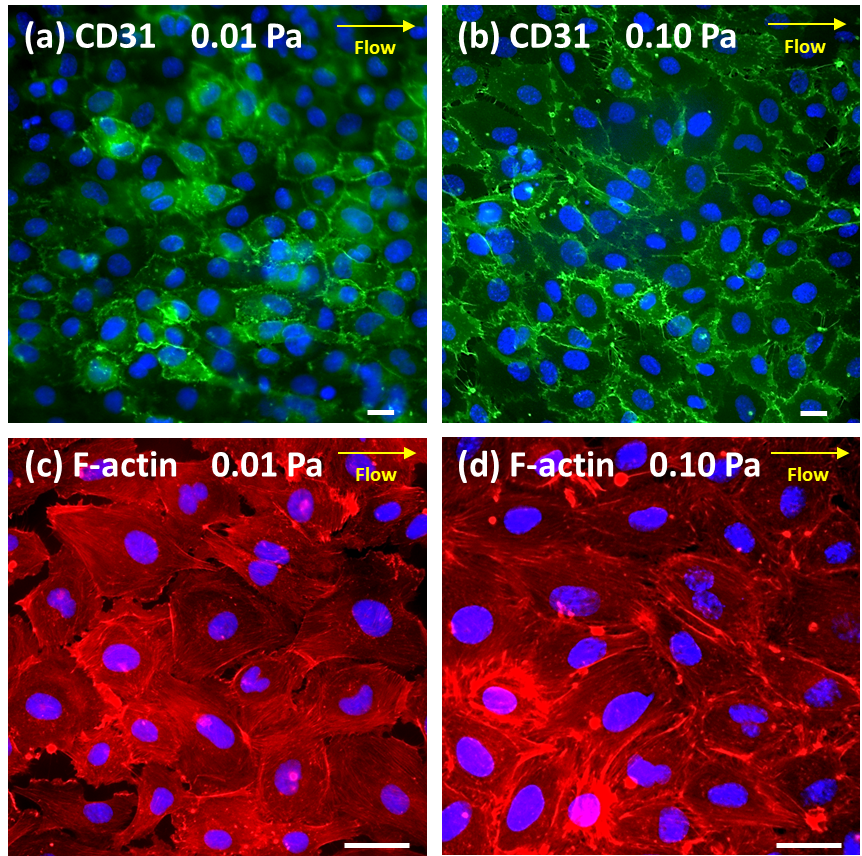


**Figure S4.** Effects of shear stress ($\tau_{w}$) on ECs. (a) and (b) CD31-stained ECs with $\tau_{w}$ of 0.01 Pa and 0.1 Pa, respectively. (c) and (d) F-actin images of ECs with $\tau_{w}$ of 0.01 Pa and 0.1 Pa, respectively. All scale bar = 20 μm.

**Supplementary Information 4 – Barrier Function of Endothelium**

Figs. S3a and S3b show BMSCs cultured for 24 days with collagen in the stroma chamber. In comparison to BMSCs cultured for 12 h (Fig. 4b), it was apparent that BMSCs were uniformly distributed in the stroma chamber during the 24-day period. The SEM images in Figs. S3c and S3d show the detailed morphological features of BMSCs developed within the collagen matrix. BMSCs were elongated with many dendrite-like protrusions through the collagen fibers. In the absence of any detectable migration of BMSCs towards the ECs, it appeared that these cells did not physically interact.

For permeability measurements, a 10x magnification objective was used to image the cross-section of the entire stroma and sinusoid chambers (Fig. S5a). The image sequences were analyzed with ImageJ and MATLAB R2020b using the dextran diffusion model previously developed by William et al.^1-3^ The diffusion model assumes that the intensity of fluorescence is proportional to the number of dextran molecules in the solution. Also, the model assumes that ${n_{Stroma \ll} n}_{Sinusoid}$ for initial flux where $n_{Stroma}$ is the number of dextran molecules in the stroma chamber and $n_{Sinusoid}$ is the number of dextran molecules in the sinusoid chamber.^1, 3, 4^ Based on the assumptions, the permeability of dextran, $P_{D}$, can be related to fluorescence intensity using the following equation:^1, 3, 5^

$P_{D}=\left( \frac{dI_{Stroma}}{dt} \right)\left( \frac{V_{Sinusoid}}{A} \right)\left( \frac{1}{I_{Sinusoid}} \right)$ Eq. S1

Where $I_{Sinusoid}$ is the original intensity of dextran in the sinusoid chamber, $I_{Stroma}$ is the intensity of dextran in the stroma chamber, $A$ is the cross-sectional area of the sinusoid vessel that was imaged, and $V_{Sinusoid}$ is the optical volume in which the fluorescence intensity is imaged. $V_{Sinusoid}$ was computed as the cross-sectional area of the sinusoid chamber, as defined in Fig. S5a, multiplied by the optical slice thickness determined by the point spread function of the imaging setup. By assuming that the optical thickness is much less than the height of the rectangular sinusoid chamber, Eq. S1 can be simplified to:

$P_{D}=\left( \frac{h}{I_{Sinusoid}} \right)\left( \frac{dI_{Stroma}}{dt} \right)$ Eq. S2

Where $h$ is the height of the sinusoid chamber and was determined experimentally. MATLAB was used to calculate $P_{D}$ from time lapse images using Eq. S2 (Fig. S5e).


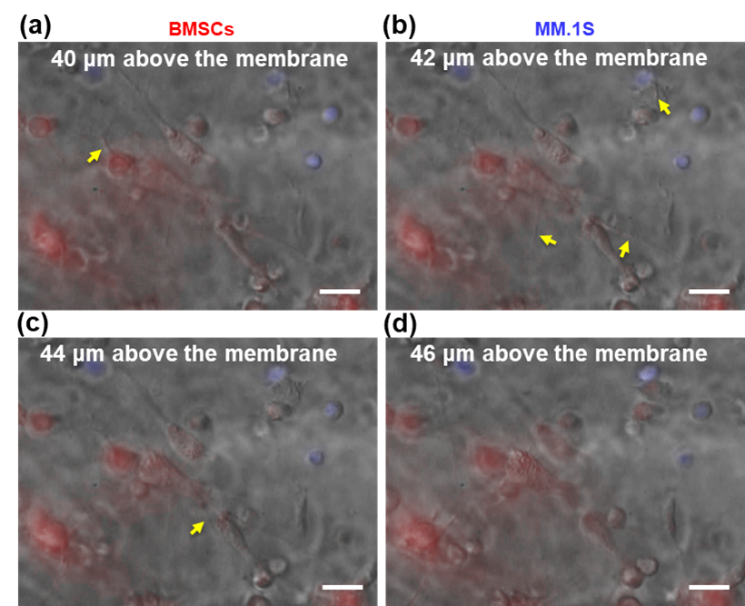


**Figure S6.** Confocal fluorescent images of BMSCs (red, CMTPX) and MM.1S (blue, Hoechst) cells in the stroma chamber after 26 h culture followed by CXCL12 added in the sinusoidal culture medium for 4 h. The yellow arrows indicate BMSCs’ extended tenacles. Scale bar = 20 μm.


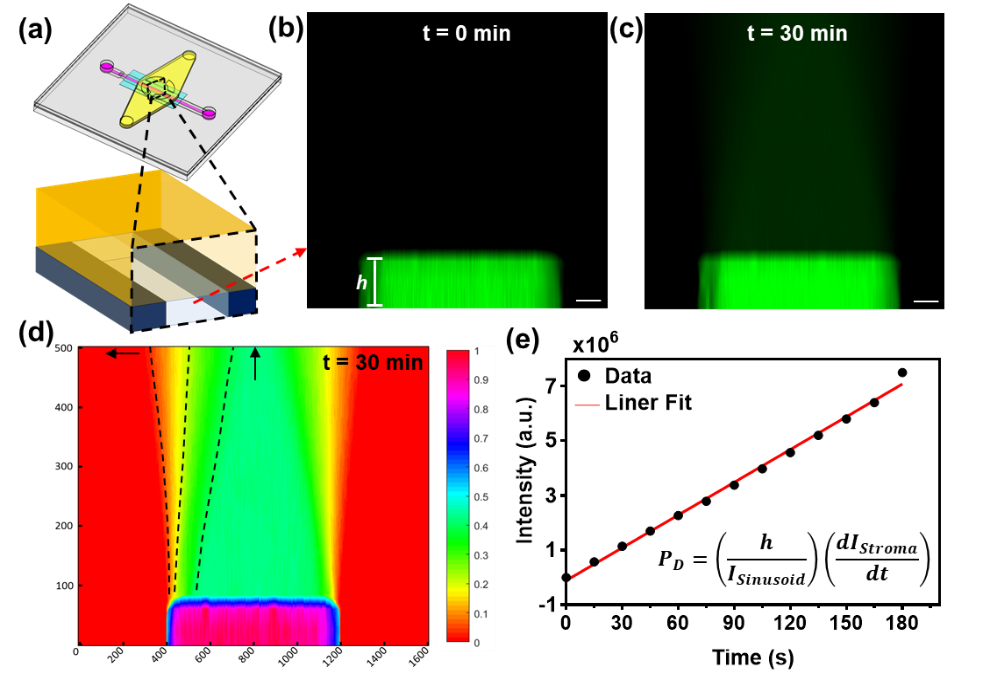


**Figure S5.** Procedures used to measure the permeability ($P_{D}$) of dextran through the endothelium constructed at the sinusoid/stroma chamber interface. (a) Schematic illustrations, showing where fluorescent time-lapse images were acquired. (b) and (c) Fluorescent mages capturing the diffusion of 70 kDa FITC-dextran at t = 0 min and t = 30 min, respectively, into the stroma chamber. Scale bar = 100 μm. (d) Spatially resolved patterns of dextran at 30 min, showing two gradient directions indicated by the black arrows in the stroma chamber, i.e., bottom to top and middle to sides. (e) The intensity of dextran in the stroma chamber plotted as a function of time and used to determine $P_{D}$ using Eq. 2.

**Supplementary Information 5 – CXCL12 Production by ECs and BMSCs**

We used ELISA (DAS00, R&D Systems) to measure CXCL12 expression of ECs and BMSCs (Fig. S7) as well as osteoblasts (hFOB cell line) for comparison. Cells were seeded at 20, 000 cells/well. Osteoblasts expressed a high level of CXCL12 with the highest level produced about at 3 days (2270 pg/mL) and then continued to decline. In contrast, ECs and BMSCs produced very small levels of CXCL12 (below 100 pg/mL). The detectable range of the ELISA kit is 156.0-10,000 pg/mL with the sensitivity of 47 pg/mL, explaining the large error bars in Fig. S7b. Also, note that these measured concentrations from ECs and BMSCs were significantly lower than 640 ng/mL, the concentration used to induce the egression of MM.1S cells.


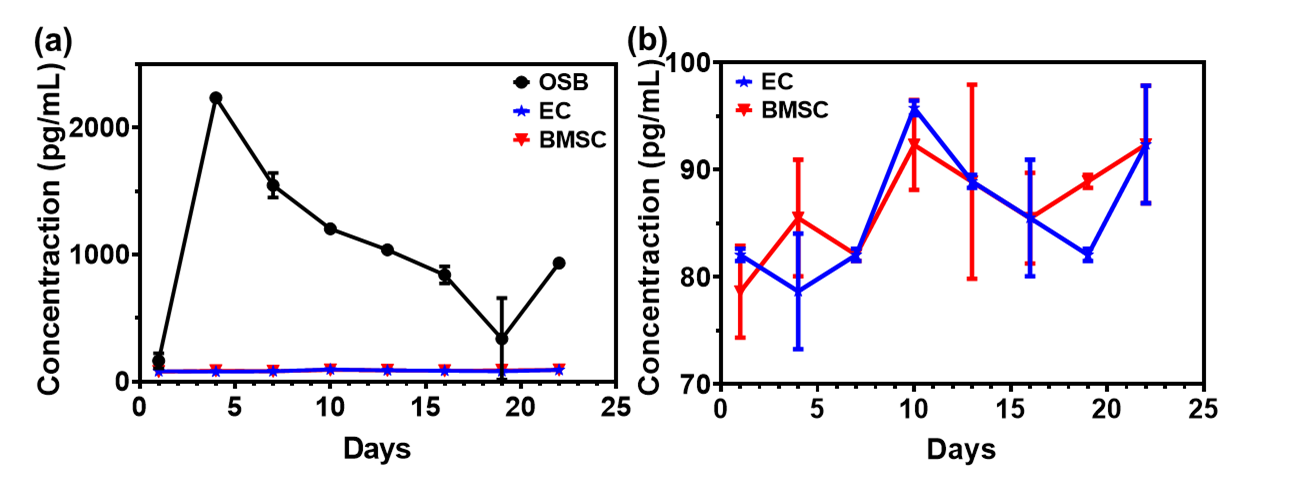


**Figure S7.** CXCL12 expressed by ECs and BMSCs in comparison to osteoblasts (OSB). Triplicates with mean±SD.

**References**

1. Polacheck, W.J., et al. Microfabricated blood vessels for modeling the vascular transport barrier. *Nat Protoc*. 14, 1425-1454 (2019).

2. Fu, B.M., et al. Determination of microvessel permeability and tissue diffusion coefficient of solutes by laser scanning confocal microscopy. *J Biomech Eng*. 127, 270-278 (2005).

3. Adamson, R.H., et al. Quantitative Laser Scanning Confocal Microscopy on Single Capillaries: Permeability Measurement. *Microcirculation*. 1, 251-265 (1994).

4. Alimperti, S., et al. Three-dimensional biomimetic vascular model reveals a RhoA, Rac1, and N-cadherin balance in mural cell-endothelial cell-regulated barrier function. *Proc Natl Acad Sci USA*. 114, 8758-8763 (2017).

5. Polacheck, W.J., et al. A non-canonical Notch complex regulates adherens junctions and vascular barrier function. *Nature*. 552, 258-262 (2017).
